# Dedifferentiated Leiomyosarcoma of the Uterus with Heterologous Elements: A Potential Diagnostic Pitfall

**DOI:** 10.1155/2012/534634

**Published:** 2012-10-17

**Authors:** Kojo R. Rawish, Oluwole Fadare

**Affiliations:** ^1^Department of Pathology, Microbiology and Immunology, Vanderbilt University School of Medicine, Nashville, TN 37232, USA; ^2^Department of Obstetrics and Gynecology, Vanderbilt University School of Medicine, Nashville, TN, USA; ^3^Department of Pathology, Microbiology and Immunology, Vanderbilt University Medical Center, 1161 21st Avenue S., Nashville, TN 37232, USA

## Abstract

Dedifferentiation is a phenomenon that is well characterized in a variety of tumors and is defined by the occurrence of a high-grade or undifferentiated tumor, typically unrecognizable regarding its line of differentiation, from a low-grade/borderline neoplasm. This phenomenon has previously been described in 2 uterine leiomyosarcomas, but both were devoid of heterologous elements. The authors describe herein a case of a dedifferentiated leiomyosarcoma of the uterus with osteoid heterologous elements, believed to be the first such reported case. The original tumor was a high-grade leiomyosarcoma with large low-grade and leiomyoma-like areas and whose constituent cells displayed intense nuclear immunoreactivity for both estrogen receptor (ER) and progesterone receptor (PR) in approximately 30% of cells. The tumor recurred six months after its resection as an undifferentiated sarcoma that was negative for smooth muscle markers, but which remained positive for ER and PR. Osteoid production was only identified in the recurrent tumor and was significant in extent therein. This case highlights the immunophenotypic changes that may occur in dedifferentiated leiomyosarcomas, and this possibility should be a consideration when an apparently undifferentiated sarcoma is identified in a patient with a history of uterine leiomyosarcoma. In our case, the expression of ER and PR provided significant supportive evidence of the uterine origin of the recurrent tumor.

## 1. Introduction

Leiomyosarcoma of the uterus is uncommon but represents the most frequently diagnosed pure sarcoma of the uterine corpus [1, 2]. The molecular events that underlie the genesis of uterine leiomyosarcomas remain largely unknown [3], but emerging lines of evidence suggest that some leiomyosarcomas, most of which are of high grade, have the capability to evolve from benign lesions or progress into lesions that are more biologically aggressive. Regarding the former, cases of “myometrial dysplasia (atypical myometrial hyperplasia)” that may represent a precursor lesion to leiomyosarcoma have been described [4], as have leiomyosarcomas that appeared to be arising directly out of leiomyomas [5–7]. Molecular and immunohistochemical lines of evidence support the derivation of some uterine leiomyosarcomas from associated leiomyoma and symplastic leiomyoma-like areas [5]. On the other end of the spectrum, biologic progression is exemplified in cases described as showing tumor dedifferentiation [8, 9], which has been described twice previously. The authors describe herein what is believed to be the first reported case of a dedifferentiated leiomyosarcoma of the uterus with osteoid heterologous elements. 

## 2. Case Presentation

A 48-year-old female underwent a supracervical hysterectomy for presumed uterine leiomyomata. Following a pathologic diagnosis of a uterine leiomyosarcoma, she underwent a bilateral salpingo-oophorectomy with full staging procedures shortly thereafter and was assigned an International Federation of Gynecology and Obstetrics stage of IA after a detailed evaluation of the resultant specimens. She underwent 4 cycles of adjuvant chemotherapy with gemcitabine and Taxotere. Six months after her hysterectomy, she underwent a followup computed tomographic scan, which revealed an 11 cm posterior pelvic mass as well as multiple intraperitoneal serosal implants. An exploratory laparotomy was performed, during which some tumor debulking was done, including a mass that was infiltrating a segment of small bowel. She was started on Adriamycin and is currently alive with disease, 8 months after her hysterectomy.

**Figure 1:**
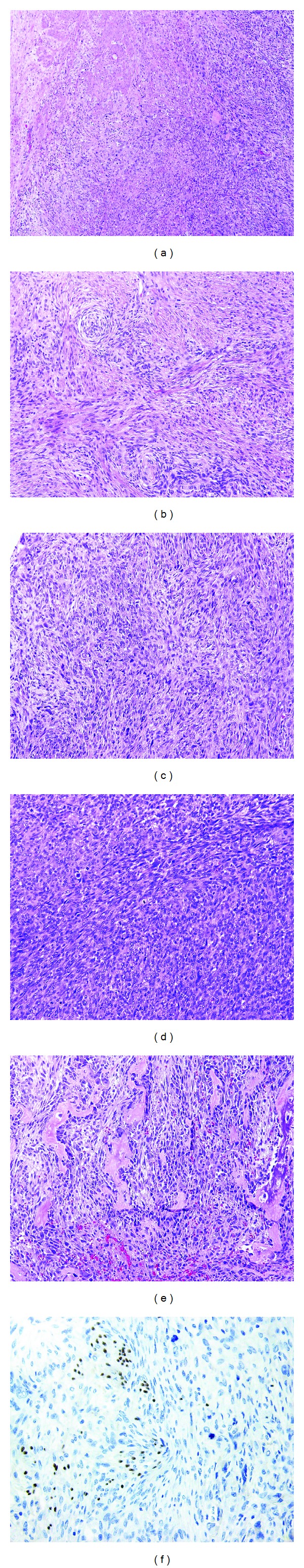
(a) Uterine tumor, areas of transition between the high-grade component (lower right field) and the “leiomyoma-like” areas (upper left field) (hematoxylin and eosin, original magnification ×100). (b) Uterine tumor, low-grade areas reminiscent of leiomyoma (hematoxylin and eosin, original magnification ×200). (c) Uterine tumor, High grade areas (hematoxylin and eosin, original magnification ×200). (d) Extrauterine tumor (hematoxylin and eosin, original magnification ×200). (e) Extrauterine tumor, showing osteoid formation (hematoxylin and eosin, original magnification ×200). (f) Extrauterine tumor, showing scattered cells with expression of the estrogen receptor (immunoperoxidase, original magnification ×200).

The hysterectomy specimen was received, morcellated, and in aggregate, measured 18 × 16.8 × 5.6 cm, and weighed 579 grams. In addition to conventional leiomyomata, there were several fragments that displayed morphologic features diagnostic of leiomyosarcoma. The latter areas showed a striking spectrum (Figures 1(a), 1(b), and 1(c)). At one end of the spectrum (representing 20% of the tumor) were areas typical of a high-grade spindle cell leiomyosarcoma, that is, a spindle cell proliferation with tumor cell (coagulative) necrosis, diffuse moderate to severe atypia, and a mitotic index of 22 mitotic figures/10 high-power fields (using the average count methods and counting 50 fields with a 40X (0.55 mm diameter) objective), Figure 1(c). Other areas that were in direct morphologic continuity with the leiomyosarcomatous areas were essentially indistinguishable from a conventional leiomyoma, since they lacked all of the aforementioned features (these areas, along with areas of hyalinization, represented approximately 60% of the tumor), Figures 1(a) and 1(b). Other areas showed “intermediate” features, in that they showed diffuse mild atypia, a mitotic index of 5 to 13 mitotic figures/10 high-power fields, and no tumor cell necrosis. No heterologous elements were identified. The tumor showed no involvement of the uterine serosa, uterine cervix, ovaries, or fallopian tubes. Immunohistochemical studies were performed on representative sections of the high-grade areas using conventional methods: paraffin slides were cut at 4 microns and baked for 15 minutes at 60°C. Slides were stained on the Leica Bond-Max platform (Leica Microsystems, Buffalo Grove, IL, USA) or the Ventana Benchmark Ultra or XT platform (Ventana Medical Systems, Tucson, AZ, USA). Deparaffinization and antigen retrieval were performed on the instrument. The primary antibody, then a secondary antibody, and then a tertiary or polymer were applied. The primary antibodies included estrogen receptor (ER, clone SP-1, prediluted, Dako, Carpinteria, CA, USA), the progesterone receptor (PR, clone 1E2, prediluted, Dako), desmin (clone DE-R-11, Leica Microsystems, prediluted, Buffalo Grove, IL, USA), alpha-actin (SMA, clone alpha-sm-1, dilution 1 : 5, Leica Microsystems), muscle-specific actin (MSA, HHF-35, prediluted, Leica Microsystems), CD34 (QBEnd/1, Leica Microsystems, Prediluted), polyclonal S100 (prediluted, Leica Microsystems), c-kit (CD117, clone YR145, prediluted, Cell Marque, Rocklin, CA, USA), h-caldesmon (clone h-CD, dilution 1; 100, Dako), and epithelial membrane antigen (EMA, clone E29, Prediluted, Cell Marque). Endogenous peroxidase was blocked using 3% hydrogen peroxide. Slides were then stained with DAB chromogen and counterstained in hematoxylin for visualization. Positive and negative controls were run in parallel as appropriate. Lesional cells displayed intense nuclear immunoreactivity for both ER and PR in approximately 30% of cells; they were diffusely positive for MSA and SMA and showed patchy immunoreactivity for desmin and h-caldesmon. CD117, CD34, S100, and EMA were negative. The debulked tissues during the third surgery consisted of segments of small bowel with serosal tumor nodules and omentum biopsies; all were diffusely involved by tumor. These deposits were a cellular fusiform to spindle cell proliferation, predominantly diffuse but also configured in storiform patterns, with tumor cell necrosis, diffuse intermediate to severe atypia, and a mitotic index of greater than 50 mitotic figures per 10 high-power fields (Figure 1(d)). This tumor also showed (in approximately 10% of the tumor volume) trabecular-patterned deposits of osteoid material, each of which was rimmed by malignant tumor cells and multinucleated osteoblastic cells, and all of which were set in a variably myxedematous background (Figure 1(e)). Immunohistochemical studies were performed on multiple blocks. Lesional cells in these areas displayed intense nuclear immunoreactivity for both ER and PR in approximately 5% of cells (Figure 1(f)); they were however negative for MSA, SMA, desmin, h-caldesmon, CD34, S100, EMA, and CD117. Given the shared expression of ER and PR between the uterine and extrauterine tumors, as well as the short interval in their clinical evolutions and discoveries, the latter was interpreted as a dedifferentiated leiomyosarcoma with heterologous (osteoid) elements.

## 3. Discussion

Dedifferentiation is a phenomenon that is well characterized in a variety of tumors and is defined by the occurrence of a high grade or undifferentiated tumor, typically unrecognizable regarding its line of differentiation, from a low-grade/borderline neoplasm, and usually with a well-defined demarcation between them [10]. In the largest series of dedifferentiated leiomyosarcomas (from all anatomic sites) reported to date (18 cases), Chen et al. [9] defined DDL as leiomyosarcomas “showing features of low-grade leiomyosarcoma associated with a discrete undifferentiated component lacking morphological or immunophenotypic features of myogenic differentiation [9]. Two of the reported cases were uterine. Heterologous osseous or chondro-osseous elements were identified in 2 of the 18 cases but in neither of the uterine cases. 

The question of whether there are any true low-grade leiomyosarcomas of the uterus is controversial, since most of these “low-grade” cases are biologically aggressive [11]. However, what is noncontroversial is that a significant subset of high-grade leiomyosarcomas display areas that are morphologically subdiagnostic of leiomyosarcoma, low grade, or even leiomyomatous [5–7]. The current case can be classified as a dedifferentiated leiomyosarcoma due to the low-grade areas (or areas that were subdiagnostic of a high-grade leiomyosarcoma within the uterine tumor), their morphologic transitions to the higher-grade regions, and the inability to demonstrate myogenic differentiation in the extrauterine lesions. Also supportive of that classification was the presence of heterologous elements, which as previously noted are within the recognized spectrum of dedifferentiated leiomyosarcoma as well as other dedifferentiated neoplasms. Although primary uterine osteosarcomas are well described [12], heterologous osteoid elements are extraordinarily rare in the uterine leiomyosarcomas. Previously reported examples of this phenomenon include 2 cases of complex tumors, reported as *mesenchymomas*, with leiomyosarcomatous, osteosarcomatous, and liposarcomatous elements [13, 14], 1 case of a mixed osteosarcoma/leiomyosarcoma [15] and 1 case of a conventional uterine leiomyosarcoma that metastasized as a high-grade sarcoma with a multitude of heterologous malignant mesenchymal elements that included osteosarcomatous, chondrosarcomatous, and liposarcomatous areas [16]. In 3 of these 4 cases, myogenic areas were clearly demonstrable either morphologically or immunohistochemically in the heterologous areas [13–15]. In the 4th case, areas of smooth muscle differentiation were focal but were still demonstrable [16]. Although the case described by Iihara et al. [8] was reported as a uterine leiomyosarcoma showing foci of dedifferentiation, smooth muscle differentiation was still demonstrable in the ostensibly dedifferentiated areas. Therefore, by strict criteria, those cases did not represent true dedifferentiation. In the current case, despite an extensive analysis, neither the morphologic features nor the immunophenotypic profile allowed the demonstration of smooth muscle differentiation.

In summary, a case of dedifferentiated leiomyosarcoma of the uterus with heterologous elements is reported. This possibility should be considered whenever a patient with a history of a resected uterine leiomyosarcoma presents with an apparently undifferentiated pleomorphic sarcoma or a sarcoma with heterologous elements at another site, and myogenic differentiation is not demonstrable in the extrauterine tumor. This case suggests that ER and PR may be useful in establishing a uterine origin for some of these cases, although their expression in the recurrent tumor may be substantially lower than in the primary tumor.
